# Rapid Adrenal Atrophy Following Excision of an Ectopic Adrenocorticotropin-Secreting Lung Carcinoid Tumor

**DOI:** 10.1210/jcemcr/luaf180

**Published:** 2025-08-22

**Authors:** Einas Mohamed, Wessam Osman, Deborah Papadopoulou, Rashpal Flora, Karim Meeran

**Affiliations:** Division of Diabetes, Endocrinology and Metabolism, Section of Endocrinology and Investigative Medicine, Imperial College London, London W12 0NN, UK; Division of Diabetes, Endocrinology and Metabolism, Section of Endocrinology and Investigative Medicine, Imperial College London, London W12 0NN, UK; National Diabetes and Endocrine Centre, Royal Hospital, Muscat 132, Oman; Division of Diabetes, Endocrinology and Metabolism, Section of Endocrinology and Investigative Medicine, Imperial College London, London W12 0NN, UK; Department of Cellular and Molecular Pathology, Hammersmith Hospital Campus, Imperial College Healthcare NHS Trust, London W12 0NN, UK; Division of Diabetes, Endocrinology and Metabolism, Section of Endocrinology and Investigative Medicine, Imperial College London, London W12 0NN, UK

**Keywords:** carcinoid, adrenal, glucocorticoid, Cushing, SST, weaning

## Abstract

Ectopic adrenocorticotropin (ACTH) secretion, a rare cause of ACTH-dependent Cushing syndrome, may be caused by neuroendocrine tumors (NETS). Postoperative hypothalamic-pituitary-adrenal (HPA) axis suppression is expected due to prolonged ACTH and cortisol overproduction. Pituitary corticotrophs are suppressed, but the adrenals are hyperplastic, and cortisol is expected to rise exuberantly after ACTH stimulation. An early postoperative short Synacthen test (SST) can therefore be unreliable as a marker of the HPA axis. Recovery of corticotrophs and adrenals is unpredictable. We report a 36-year-old woman with severe Cushing syndrome. Biochemical evaluation confirmed ACTH-dependent hypercortisolism. Inferior petrosal sinus sampling was consistent with an ectopic ACTH source, and imaging revealed a 10-mm tracer-avid pulmonary lesion. Surgical excision confirmed an ACTH-secreting atypical carcinoid tumor. Postoperatively, the patient exhibited profound ACTH and cortisol deficiency and was discharged on once-daily prednisolone replacement. Seven weeks after surgery, she had an unexpectedly flat SST with undetectable cortisol levels, suggesting rapid adrenal atrophy. Gradual HPA recovery was documented with slow prednisolone withdrawal over the following year. This case demonstrates rapid adrenal atrophy and encouragingly early full HPA-axis recovery. There is no cutoff value below which adrenal recovery is impossible. A postoperative SST may hinder glucocorticoid-tapering strategies.

## Introduction

Ectopic adrenocorticotropin (ACTH) secretion is responsible for approximately 10% of ACTH-dependent Cushing presentations. It typically originates from bronchopulmonary neuroendocrine tumors, and is usually managed by surgical excision. The legacy assumption that postoperative adrenals remain hyperplastic and therefore Synacthen-responsive can be misleading. Abrupt ACTH withdrawal may therefore trigger rapid cortical atrophy and confound steroid-tapering decisions.

## Case Presentation

A 36-year-old woman gave birth to a healthy baby and subsequently noticed substantial weight gain. She was diagnosed with postpartum depression and prescribed citalopram, attributing her weight gain to her depression, the stress of her studies, and anxiety due to the COVID-19 pandemic. Despite exercising regularly and attempting various diets, she realized that her weight gain was concentrated around her shoulders and abdomen, and her face began to appear rather round. She also noticed excessive hair growth in different areas and began to feel weak, with her muscles failing to support her, causing her to stumble. She observed some purple striae around her abdomen and found it easy to bruise. A medical evaluation at that time revealed she was hypertensive and prediabetic, although she had not had diabetes during her pregnancy. The patient suspected Cushing syndrome herself, as she works as a physiotherapist.

Physical examination confirmed typical signs of Cushing syndrome, including a rounded moon face, abdominal purple striae associated with centripetal obesity, proximal myopathy, a dorsocervical fat pad, and thinning scalp hair.

## Diagnostic Assessment

Her initial laboratory results indicated a late-night salivary cortisol level of 15.3 nmol/L (5.1 ng/mL) (reference range, SI <2.6 nmol/L [2.2-5.1 ng/mL]), a late-night salivary cortisone level of 63.3 nmol/L (20.9 ng/mL) (reference range, SI <18 nmol/L [<5 ng/mL]), and a urinary free cortisol excretion of 1095 nmol/24 h (396 μ/24 h) (reference range, SI 0-164 nmol/L/24 h [<50 μg/24 h]). Serum cortisol was measured at 488 nmol/L (17.7 μg/dL) (reference range, SI 138-635 nmol/L [5-23 μg/dL]), and ACTH was recorded at 81.8 pg/mL (18.0 pmol/L) (reference range, 5-50 pg/mL [SI 1.6-13.93 pmol/L]). The remainder of her hormonal panel fell within the normal range. Her overnight dexamethasone suppression test revealed failure of cortisol suppression with a post–dexamethasone cortisol level of 499 nmol/L (18.1 μg/dL) (normal response <50 nmol/L [<18.1 ng/mL]), with an adequate dexamethasone absorption level of 3.0 nmol/L (1.18 ng/mL) (positive absorption if >3 nmol/L [<1.18 ng/mL]).

Results of the inferior petrosal sinus sampling (IPSS) are displayed in Table 1. The prolactin levels confirm that only the left side has been adequately cannulated, so that the right-sided ACTH levels may be uninterpretable. There was no difference between the pituitary ACTH (on either side) and the peripheral ACTH consistent with ectopic ACTH. Typically, in patients with pituitary-dependent Cushing disease, there is at least a 2-fold gradient between the pituitary and peripheral ACTH levels [1].

**Table 1.** Inferior petrosal sinus sampling via 10 mcg of intravenous desmopressin administered at 0 minutes

Time, min	ACTH left	ACTH right	ACTH peripheral	Prolactin left	Prolactin right	Prolactin peripheral
−7	101.0 ng/L (101.0 pg/mL)	95.1 ng/L (95.1 pg/mL)	81.5 ng/L (81.5 pg/mL)	814 mLU/L (38.40 ng/mL)	194 mLU/L (9.15 ng/mL)	184 mLU/L (8.68 ng/mL)
−5	92.3 ng/L (92.3 pg/mL)	92.3 ng/L (92.3 pg/mL)	84.1 ng/L (84.1 pg/mL)	1005 mLU/L (47.41 ng/mL)	184 mLU/L (8.68 ng/mL)	189 mLU/L (8.92 ng/mL)
−2	96.1 ng/L (96.1 pg/mL)	90.1 ng/L (90.1 pg/mL)	79.1 ng/L (79.1 pg/mL)	1188 mLU/L (56.04 ng/mL)	188 mLU/L (8.87 ng/mL)	183 mLU/L (8.63 ng/mL)
0	91.9 ng/L (91.9 pg/mL)	92.9 ng/L (92.9 pg/mL)	78.0 ng/L (78.0 pg/mL)	780 mLU/L (36.79 ng/mL)	189 mLU/L (8.92 ng/mL)	179 mLU/L (8.44 ng/mL)
2	107.0 ng/L (107.0 pg/mL)	94.6 ng/L (94.6 pg/mL)	92.3 ng/L (92.3 pg/mL)	1017 mLU/L (47.97 ng/mL)	196 mLU/L (9.25 ng/mL)	188 mLU/L (8.87 ng/mL)
5	99.7 ng/L (99.7 pg/mL)	102.0 ng/L (102.0 pg/mL)	98.7 ng/L (98.7 pg/mL)	709 mLU/L (33.44 ng/mL)	199 mLU/L (9.39 ng/mL)	193 mLU/L (9.10 ng/mL)
7	106.0 ng/L (106.0 pg/mL)	96.9 ng/L (96.9 pg/mL)	87.7 ng/L (87.7 pg/mL)	657 mLU/L (30.99 ng/mL)	194 mLU/L (9.15 ng/mL)	180 mLU/L (8.49 ng/mL)
10	94.8 ng/L (94.8 pg/mL)	101.0 ng/L (101.0 pg/mL)	97.4 ng/L (97.4 pg/mL)	622 mLU/L (29.34 ng/mL)	190 mLU/L (8.96 ng/mL)	187 mLU/L (8.82 ng/mL)

ACTH normal range reference range is 9.0 to 51.0 ng/L (14.0-51.0 pg/mL), and prolactin is less than 25 ng/mL (<530 mLU/L) in adult nonpregnant individuals, and less than 20 ng/mL (<424 mLU/L) in male adults.

Abbreviation: ACTH, adrenocorticotropin.

**Figure 1.**
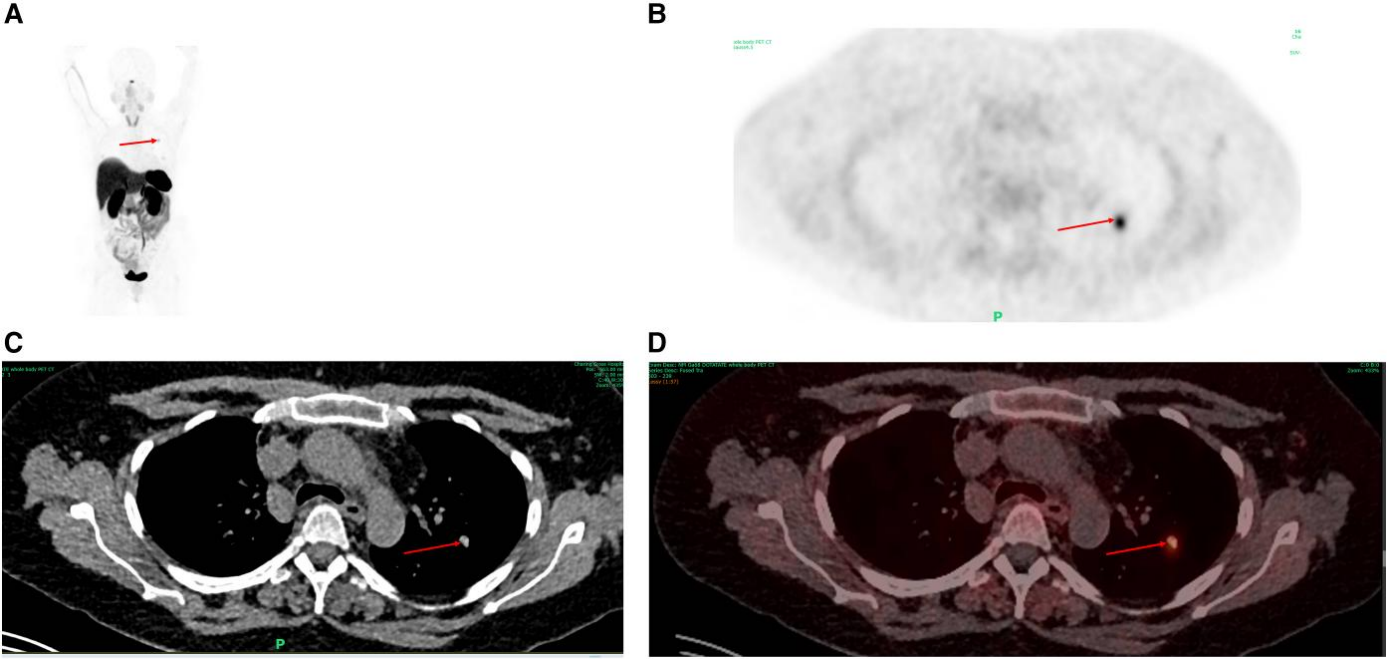
A, Maximum intensity projection of gallium68-DOTATATE scan; B, axial positron emission tomography; C, axial computed tomography; and D, fused image, with arrows pointing at the culprit lesion in all views.

A search for the ectopic source was undertaken using a gallium 68 (68-Ga) 1,4,7,10-tetraazacyclododecane-1,4,7,10-tetraacetic acid (DOTA)-octreotate (68-Ga-DOTATATE) whole-body computed tomography–positron emission tomography scan. This revealed a 10-mm lesion in the upper left lobe of the lung with moderate tracer-uptake avidity and a maximum standardized uptake value of 4.2, with no other lesions anywhere above or below the diaphragm, in keeping with a carcinoid tumor (Fig. 1).

## Treatment

**Figure 2.**
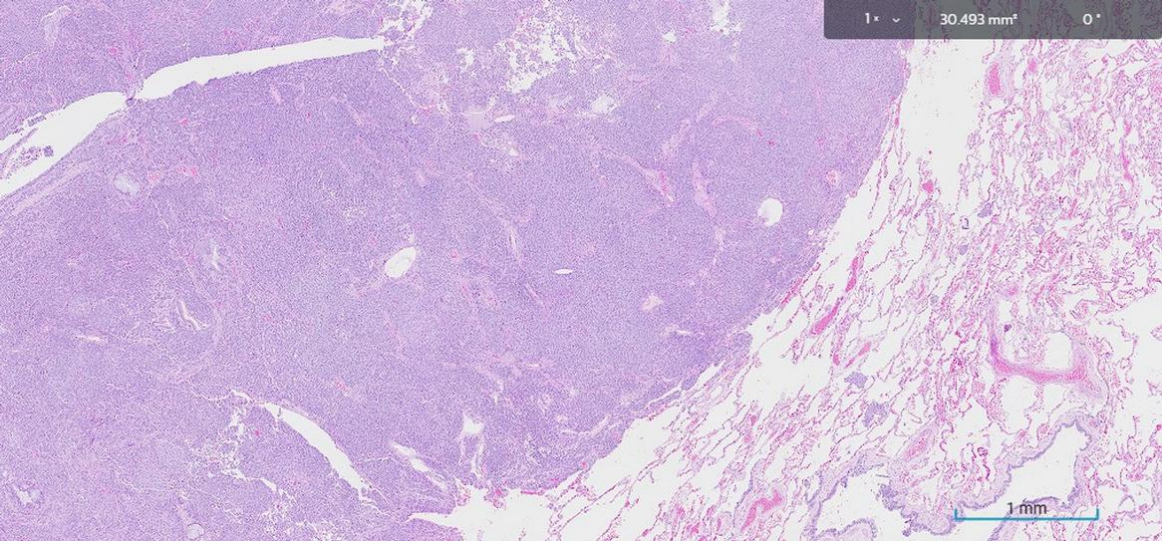
Histopathology using hematoxylin and eosin staining at ×1 magnification revealed an atypical carcinoid tumor with a nested growth pattern; the mitotic count is less than 1 per 2 mm².

**Figure 3.**
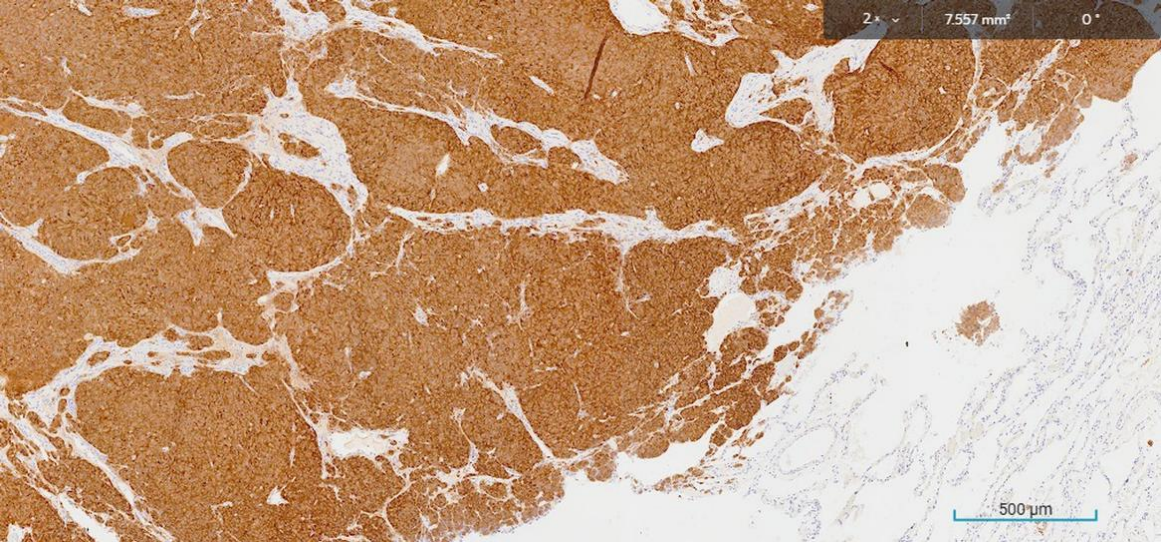
Immunohistochemistry showed expression of synaptophysin.

**Figure 4.**
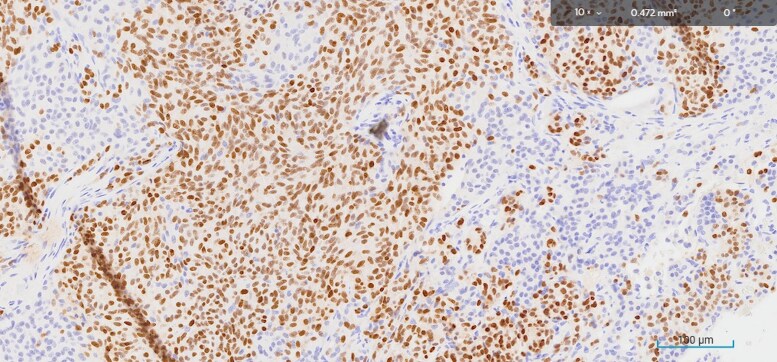
Immunohistochemistry showed expression of patchy thyroid transcription factor-1.

**Figure 5.**
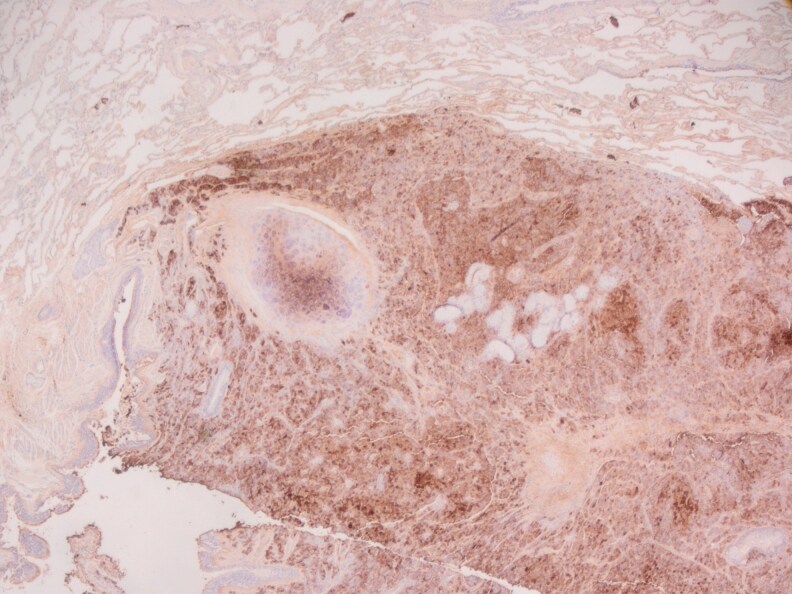
Adrenocorticotropin immunohistochemistry showing diffuse and strong staining.

A 14-mm lesion was excised from the left lung in early 2024. The tissue histopathology revealed an atypical carcinoid tumor on hematoxylin and eosin staining (Fig. 2) expressing synaptophysin (Fig. 3), and patchy thyroid transcription factor-1 (Fig. 4), supporting a diagnosis of carcinoid tumor of pulmonary origin. Diffuse and strong ACTH was also demonstrated with ACTH immunohistochemistry (Fig. 5).

## Outcome and Follow-up

The patient’s immediate postoperative cortisol level fell from 488 nmol/L (17.7 μg/dL) (preoperatively) to 34 nmol/L (1.2 μg/dL) 72 hours postoperatively. Her postoperative aldosterone level was 90 pmol/L (3.24 ng/dL) (reference range, 90-700 pmol/L [3.24-25.2 ng/dL]), renin was 0.6 nmol/L/h (0.6 ng/mL/h) (reference range, 0.5-3.5 ng/mL/h), ACTH was less than 5 pg/mL, sodium was 140 mmol/L (reference range, 135-145 mmol/L), and potassium was 4.2 mmol/L (reference range, 3.5-5.0 mmol/L). She was discharged initially on once-daily prednisolone 8 mg and once-daily rivaroxaban 10 mg. Rivaroxaban was stopped 3 months postoperatively, and the dose of prednisolone was slowly reduced to 5 mg daily 7 weeks postoperatively. She was also given oxycodone to be used as needed for her postoperative pain. Her SST on prednisolone 5 mg daily revealed a complete absence of response to Synacthen 2 months postoperatively (Table 2).

**Table 2.** Short Synacthen test showing flat response

Time	Cortisol	ACTH
T = 0 min	<28 nmol/L	<5 ng/L
T = +30 min	<28 nmol/L	—
T = +60 min	<28 nmol/L	—

Cortisol normal range is 6 to 18.4 μg/dL (165.5-507.6 nmol/L), and ACTH is 5 to 50 ng/L (2.2-10.56 pmol/L).

Abbreviation: ACTH, adrenocorticotropin.

The absence of ACTH (<5 ng/L) for 7 weeks has resulted in remarkable and complete atrophy of the adrenal glands, which were unable to respond to Synacthen. These results were unexpected, as we anticipated a characteristically exaggerated response. Her 8-hour prednisolone level appeared sufficient at 21 μg/L (reference range at 480 minutes, 15-25 μg/L). We advised her to continue 5 mg prednisolone once daily with the aim of starting a gradual weaning over the summer, recognizing that the only way to revive the hypothalamic-pituitary-adrenal (HPA) axis is to underrun her glucocorticoid (GC) replacement. Some guidelines suggest that the dose should not be reduced with very low cortisol levels, but persistent prednisolone replacement would simply cause perpetual adrenal suppression. We therefore disregarded the flat SST result and slowly reduced the prednisolone dose using a modified protocol applied at Imperial College Healthcare National Health Service Trust [2], and also recommended by the National Institute for Health and Care Excellence (NICE) [3]. The patient was keen to proceed with weaning despite experiencing GC withdrawal syndrome (GWS).

Seven months after surgery, she had successfully reduced her dose to 3 mg of prednisolone daily. A repeat SST with a prednisolone day curve at 3 mg was carried out as shown in Table 3. Her post–8-hour prednisolone level was 15 μg/L, which we deemed acceptable for continuing the weaning process. The patient enthusiastically continued the weaning for 4 months, when she returned for repeat tests while on 2 to 3 mg of prednisolone, yielding the SST results in Table 4. These results clearly indicated incipient HPA recovery. The patient was very encouraged by her results, and she herself suggested weaning her prednisolone even more rapidly, as she no longer experienced substantial GWS. Her last dose of prednisolone was taken just under 1 year after her surgical treatment.

**Table 3.** Short Synacthen test results on 3 mg of prednisolone daily

Time	Cortisol	ACTH
T = 0 min	29 nmol/L	8.7 pg/mL
T = +30 min	108 nmol/L	—
T = +60 min	147 nmol/L	—

Cortisol normal range is 6 to 18.4 μg/dL (165.5-507.6 nmol/L), and ACTH is 5 to 50 pg/mL (SI, 1.6-13.93 pmol/L).

Abbreviation: ACTH, adrenocorticotropin.

**Table 4.** Short Synacthen test on 2 to 3 mg of prednisolone daily

Time	Cortisol	ACTH
T = 0 min	197 nmol/L	18.3 ng/L
T = +30 min	307 nmol/L	—
T = +60 min	373 nmol/L	—

Cortisol normal range is 6 to 18.4 μg/dL (165.5-507.6 nmol/L), and ACTH is 5 to 50 ng/L (2.2-10.56 pmol/L).

Abbreviation: ACTH, adrenocorticotropin.

## Discussion

During the first weeks after pituitary surgery, the SST is unreliable because ACTH falls well before adrenal atrophy and cortisol deficiency become apparent. An ostensibly normal result obtained immediately after total hypophysectomy, therefore, represents a false negative [4]. The recommendation is to defer the test for a minimum of 2 weeks, during which time adrenal atrophy will have manifested if the patient is ACTH deficient, and the consequent loss of ACTH will be evident in a genuine inability to respond to Synacthen [5]. The renin-aldosterone system was not affected, as evidenced by the normal aldosterone of 90 pmol/L (3.24 ng/dL) and renin 0.6 nmol/L/h (0.6 ng/mL/h), as mentioned earlier.

In patients with ectopic ACTH, the adrenal glands are expected to be hyperplastic and elicit an exuberant response to Synacthen both before and immediately after surgery. The complete absence of ACTH following removal of the ectopic source leads to atrophy of the adrenal glands, and our case demonstrates that complete atrophy can occur as rapidly as 7 weeks post surgery. All patients with ectopic ACTH who undergo surgical intervention should have a functional pituitary gland, enabling the safe weaning of GC therapy once the corticotrophs have undergone recovery. However, many clinicians mistakenly believe that all patients with a flat Synacthen test should remain on lifelong replacement therapy, which our case unequivocally demonstrates is incorrect. The postoperative atrophy of the adrenal glands may necessitate a slower weaning process, but patients should still be encouraged to wean despite experiencing daunting symptoms of GC withdrawal [3]. When endogenous cortisol levels are detectable (cortisol > 25 nmol/L [(∼ 0.9 μg/dL]), every attempt should be made to wean the patient, as demonstrated in a recent respiratory study [6].

In one retrospective study, 82% of patients with ectopic Cushing syndrome (ECS) regained HPA function within 5 years, compared to 58% in Cushing disease and 38% in adrenal Cushing syndrome [7]. It is reasonable to anticipate that all patients with a single normal adrenal gland, thereby maintaining an intact HPA axis, will demonstrate successful weaning. The same should apply to ectopic ACTH, during which all components of the HPA axis would be intact. This suggests that GWS may discourage clinicians from weaning properly. However, a higher success rate can be achieved with appropriate efforts and effective explanations to patients regarding the importance of weaning. Some patients who have pituitary surgery might have a damaged HPA axis, so one would need to be more cautious with steroid withdrawal. Patients weaned using a prednisolone protocol [2] may be more successful than those on a hydrocortisone one [8].

With appropriate weaning, success should be achievable in all ECS and adrenal Cushing cases [7]. One of the reasons patients fail to be weaned successfully is due to clinicians’ reliance on the Synacthen test to guide withdrawal. However, this approach has been demonstrated to be counterproductive [5]. Surprisingly, our patient exhibited no response to Synacthen and the Endocrine Society and European Society of Endocrinology joint guidelines [9], suggesting that the patient's GC-replacement regimen should not be reduced. In contrast, we adopted a different approach and persisted with the patient's weaning process, employing a prednisolone-weaning protocol [2]. Since many patients with primary adrenal failure are well maintained on prednisolone 3 mg once daily [10], performing an SST on a dose of 3 mg or more will be misleading [11]. The equivalent dose of hydrocortisone is 20 mg as prednisolone is 7 times more potent than hydrocortisone [12, 13]. Additionally, a morning serum cortisol of greater than 237 nmol/L (8.6 μg/dL) on the Roche Cortisol-II assay reliably predicts HPA axis recovery, providing a rapid, cost-effective screening method with 100% specificity and potential resource savings [14].

Despite her complaints regarding GWS, the patient consented to a continued reduction in prednisolone dosage as her cushingoid features gradually resolved. Distinguishing between true adrenal insufficiency and GWS remains clinically challenging, given the complex neuroendocrine adaptations following chronic hypercortisolism [15].

Despite her slow yet encouraging recovery, our patient agreed to wean herself off prednisolone completely. Her only challenge was overcoming the GWS experienced during the weaning process. After a thorough explanation of the significance of “underrunning” in stimulating the adrenal axis, the patient persevered through the symptoms of GWS and reduced the dosage. The clinical distinction remains challenging due to the complex mechanisms behind GWS (Table 5), which are not fully understood [16].

**Table 5.** Glucocorticoid withdrawal syndrome vs adrenal insufficiency after surgical treatment of Cushing syndrome [10]

Comparison of GC withdrawal syndrome and postoperative adrenal insufficiency		
Category	GC withdrawal syndrome	Adrenal insufficiency
Diagnosis	Clinical symptoms in appropriate context (eg, following prolonged high-dose prednisolone treatment for polymyalgia)	Biochemical evidence of low basal serum cortisol and/or inadequate response to cosyntropin stimulation
Underlying mechanisms	Incomplete understanding; involves possible upregulation of inflammatory cytokines and prostaglandins even in presence of adequate levels of cortisol	HPA axis downregulation with subphysiologic GC replacement. Always has true deficiency of cortisol
Clinical characteristics	Fatigue, diffuse myalgias, arthralgias, and weakness Not life-threatening	Fatigue, malaise, anorexia, diffuse myalgias, and arthralgias (similar to GWS), but with more nausea, vomiting hypotension, hyponatremia, and hyperkalemia Possible adrenal crisis with hypotension and shock
Management	Patient reassurance and counseling on postoperative recovery Slow GC taper or continue same dose until patient is ready to resume taper. When using protocol, stay on same week for ∼2 wk to slow taper. A temporary dose increase to mitigate symptoms is risky, as it will likely suppress HPA axis	Careful assessment of physiologic GC needs. Increase GC dose for intercurrent illness and use injectable GC when oral intake is not possible

Abbreviations: GC, glucocorticoid; GWS, glucocorticoid withdrawal syndrome; HPA, hypothalamic-pituitary-adrenal.

In conclusion, our case highlights an unusually rapid onset of adrenal atrophy following curative surgery in ECS. In all patients with steroid-induced adrenal insufficiency, irrespective of the cause (exogenous steroids or endogenous Cushing syndrome of any type), a concerted effort should be made to wean them off GCs gradually. This applies even to modern pituitary surgery, in which most of the normal pituitary is spared. The only exception to this is in cases of total hypophysectomy, where the surgical removal of the entire pituitary gland has occurred, and weaning should not be attempted [11].

In patients who have not undergone pituitary surgery, the sole factor limiting the reduction of GCs is GWS. We strongly advocate for patients to follow a fixed prednisolone-weaning protocol. The rate of reduction should be guided by patient symptoms rather than SST values [17]. We believe SSTs should be used only when patients are on less than 3 mg prednisolone and can be used to reassure patients experiencing GWS [14].

## Learning Points

- Adrenal hyperplasia may regress to complete atrophy as early as 7 weeks after resection of an ectopic ACTH-secreting tumor.
- An early flat SST is misleading, as it signals transient ACTH deprivation, not permanent primary adrenal failure.
- Protocol-driven, symptom-guided GC tapering—rather than test-based inertia—is essential for HPA-axis reactivation.
- Dynamic adrenal testing should not be conducted even if prednisolone dose is greater than 3 mg/day (or morning cortisol > 237 nmol/L [8.6 μg/dL]) to prevent misinterpretation.

## Contributors

Joint first authorship is shared equally between E.M. and W.O. E.M. was the first person to see the patient and suspect the diagnosis and reviewed the patient several times. W.O. and D.P. also reviewed the patient regularly and undertook the IPSS. K.M. and D.P. were involved in diagnosis and management, the histopathology section was completed, and the preparation of histology images were done by R.F. All authors reviewed and approved the final draft.

## Data Availability

Data sharing is not applicable to this article as no data sets were generated or analyzed during the current study.

## Abbreviations

ACTH: adrenocorticotropin
ECS: ectopic Cushing syndrome
GC: glucocorticoid
GWS: glucocorticoid withdrawal syndrome
HPA: hypothalamic-pituitary-adrenal
IPSS: inferior petrosal sinus sampling
SST: short Synacthen test
